# Extracellular Vesicle-Associated miR-222-3p and miR-186-5p as Potential Hypoxic Markers in Canine Osteosarcoma: A Preliminary In Vitro Study

**DOI:** 10.3390/ani16081265

**Published:** 2026-04-20

**Authors:** Raffaella De Maria, Manuela Poncina, Sara Divari, Lorenza Parisi, Sonia Capellero, Luiza Cesar Conti, Eugenio Mazzone, Federica Fratini, Luca Aresu, Lorella Maniscalco

**Affiliations:** 1 Department of Veterinary Sciences, University of Turin, 10095 Grugliasco, Italy; manuela.poncina@unito.it (M.P.); sara.divari@unito.it (S.D.); luiza.cesarconti@unito.it (L.C.C.); eugenio.mazzone@unito.it (E.M.); luca.aresu@unito.it (L.A.); lorella.maniscalco@unito.it (L.M.); 2 Candiolo Cancer Institute, Fondazione del Piemonte per l’Oncologia IRCC, Istituto di Ricovero e Cura a Carattere Scientifico (FPO-IRCCS), 10060 Candiolo, Italy; sonia.capellero@unito.it; 3 Department of Neuroscience, Italian National Health Institute, 00161 Rome, Italy; federica.fratini@iss.it

**Keywords:** extracellular vesicles, microRNAs, hypoxia, biomarkers, comparative oncology

## Abstract

The hypoxic microenvironment plays a critical role in the progression of canine osteosarcoma (OSA) by promoting different cellular responses, including the release of extracellular vesicles (EVs). Given the clinical aggressiveness of canine OSA, the aim of this study was to evaluate the miRNAome profile in EVs released in vitro by four canine OSA cell lines under hypoxic conditions. In particular, for this study we used two commercial canine osteosarcoma cell lines (D17 and D22) and two primary osteosarcoma cell lines obtained in our laboratory (Penny and Wall). D17, D22, Penny, and Wall cell lines were cultured under normoxic and hypoxic conditions (200 µM CoCl_2_) for 24 h. EVs were isolated by size-exclusion chromatography and characterized by nanoparticle tracking analysis and Western blotting. miRNAs extracted from EVs were then sequenced and analyzed using bioinformatics approaches. The most representative miRNAs were identified and validated by qPCR using the miRCURY LNA miRNA PCR assay. miRNome profiling identified 233 miRNAs differentially expressed in EVs across all analyzed cell lines. Among these, 94 miRNAs were detected exclusively under hypoxic conditions. From this subset, 43 miRNAs were selected for further validation by qPCR. The qPCR results showed that miR-222-3p and miR-186-5p were significantly downregulated in the Wall cell line under hypoxia (*p* ≤ 0.05). TargetScan and pathway enrichment analyses demonstrated that miR-186-5p regulates target genes involved in different cellular processes. In human osteosarcoma, low serum levels of miR-222-3p are associated with poor prognosis, while miR-186-5p is recognized as a key hypoxia-responsive miRNA. Collectively, these results suggest the potential of EV-associated miRNAs as biomarkers in canine OSA and support their relevance in translational and comparative oncology.

## Simple Summary

Canine osteosarcoma is a highly aggressive bone cancer that primarily affects dogs and is often associated with a poor prognosis. One of the key drivers of tumor progression is reduced oxygen availability within the tumor microenvironment, a condition known as hypoxia. Under low-oxygen conditions, tumor cells release extracellular vesicles (EVs) that carry biologically active cargo and can influence the behavior of surrounding cells. The aim of this study was to investigate how hypoxia affects the epigenetic information conveyed by EVs released from canine osteosarcoma cells. We analyzed four canine osteosarcoma cell lines and compared EVs produced under normoxic and hypoxic conditions. Our results showed that hypoxia alters the epigenetic cargo of EVs and is associated with reduced levels of two specific microRNAs. These microRNAs are implicated in cancer progression and cellular adaptation to hypoxia in human bone tumors. Overall, this study advances our understanding of how canine osteosarcoma adapts to hypoxic conditions and supports the potential use of EV-associated microRNAs as biomarkers. These findings may contribute to improved diagnostic and monitoring strategies in both veterinary and human oncology.

## 1. Introduction

Osteosarcoma (OSA) is a highly malignant tumor characterized by neoplastic mesenchymal cells producing an immature osteoid matrix. It is the most common primary bone malignancy in both children and dogs, sharing key features across species, including microscopic metastatic dissemination at diagnosis, similar responses to surgery and chemotherapy, and dysregulation of multiple molecular pathways [1]. Despite aggressive therapy, survival has not improved over the past 15 years, with reported mortality rates of 30–40% in children and >90% in dogs [2]. In both species, OSA most commonly arises in the long bones of the appendicular skeleton, most often the distal femur, proximal tibia, and proximal humerus, while flat bones and the spine are rarely affected. In humans, peak incidence occurs during adolescence, suggesting an association with growth-related factors [3]. In contrast, canine OSA develops in skeletally mature, large- and giant-breed dogs, with ~75% of cases affecting the appendicular skeleton, most frequently the distal radius and proximal humerus [1].

These shared clinical and molecular features, together with the higher natural incidence in dogs, make appendicular OSA a valuable translational model [2,4]. However, survival remains limited, and reliable prognostic biomarkers are still lacking [1]. For these reasons, it is essential to identify new therapeutic strategies by investigating novel molecular indicators that could provide both prognostic value and potential therapeutic targets to improve patient outcomes.

MicroRNAs (miRNAs) are ~22-nt non-coding RNAs that post-transcriptionally regulate a large fraction of the genome, influencing development, metabolism, apoptosis, and cancer occurrence [5]. Extracellular vesicles (EVs), including exosomes and microvesicles, are stable carriers of miRNAs and other cargo detectable in multiple body fluids, mediating intercellular communication and contributing to tumor progression and the formation of pre-metastatic niches [6]. In OSA, EV-omics studies and circulating miRNA profiles are emerging as promising minimally invasive tools, particularly in a comparative oncology setting [7,8], as well as in translational xenograft models defining tumor-derived exosomal RNA signatures [9]. Among miRNAs implicated in OSA biology, miR-186-5p has been reported as a tumor suppressor in human OSA, inhibiting proliferation, migration, and invasion by targeting genes such as FOXK1 and TBL1XR1 [10]. Conversely, miR-222-3p is frequently overexpressed in human OSA tissues and serum and promotes migration and invasion partly via suppression of TIMP3 [11]. Although plasma miRNA signatures have shown prognostic value in canine OSA (e.g., miR-214-3p and panels of circulating miRNAs), exosomal microRNAs (exomiRs) remain underexplored in this species. Nevertheless, EV-miR-based detection is feasible in canine oncology, and EV-associated miR-222-3p has been detected in dogs with hematologic malignancies [12], supporting the translational applicability of these assays [13].

Hypoxia is a key component of the heterogeneity of solid tumors and is a consequence of the high tumor cell proliferation rate and the abnormal structure of the tumor vasculature, driving tumor progression, chemoresistance, and metastasis through activation of HIF-1α and VEGF pathways [14]. To induce hypoxic conditions in vitro, cell lines are typically grown in hypoxia chambers, which better reproduce physiological hypoxic conditions [15]. However, in several studies, chemical treatment of cell lines with cobalt chloride (CoCl_2_), a compound that induces HIF-1α accumulation, is used as an alternative method [16]. We recently demonstrated that OSA cell lines under CoCl_2_ treatment accumulate HIF-1α in the nucleus, with activation of hypoxia-related gene transcription, suggesting effective biological effects of HIF-1α on mRNA regulation [17]. Based on this evidence, we hypothesized that under the same conditions, OSA cell lines could also be induced to modulate miRNA regulation.

Thus, the aim of this study was to characterize EV-associated miRNA profiles in canine appendicular OSA cell lines under normoxic and CoCl_2_-induced hypoxic conditions and to investigate their potential as biomarkers and contributors to OSA pathogenesis.

## 2. Materials and Methods

### 2.1. Cell Culture Conditions

Four canine osteosarcoma (OSA) cell lines were used: D17 (ATCC^®^ CCL-183™), D22 (ATCC^®^ CRL-6250™), and two previously characterized and validated primary canine OSA cell lines, Penny and Wall [18]. D17 and D22 cell lines were cultured in DMEM (Gibco, Thermo Fisher Scientific, Waltham, MA, USA) medium supplemented with 10% fetal bovine serum (FBS) (Gibco), 1% L-glutamine, 100 μg/mL penicillin (Gibco), and 100 μg/mL streptomycin (Gibco) while Penny and Wall were grown in IMDM (Gibco) supplemented with 10% fetal bovine serum (FBS) (Gibco), 1% L-glutamine, 100 μg/mL penicillin (Gibco), and 100 μg/mL streptomycin (Gibco), in a humidified incubator at 37 °C with 5% CO_2_. Forty-eight hours prior to extracellular vesicle collection, cells were maintained in serum-free culture medium to avoid the presence of bovine-derived EVs.

### 2.2. Hypoxia Induction

A total of 2200 cells per well from each canine OSA cell line (D17, D22, Penny, Wall) were seeded in 96-well CellCarrier Ultra plates (PerkinElmer, Waltham, MA, USA) and exposed to chemically induced hypoxia using cobalt chloride (CoCl_2_; Sigma-Aldrich, St. Louis, MO, USA—Cat. No. 15862) at a final concentration of 200 µM for 24 h, as previously described [14]. Cells cultured in serum-starved medium without CoCl_2_ served as normoxic controls for each time point. All treatments were performed in triplicate wells, and each experiment was independently repeated three times.

### 2.3. Extracellular Vesicle Isolation and Characterization

Following hypoxia treatment, conditioned media from each cell line and experimental condition were collected. Exosomes were isolated using the Exo-spin™ Exosome Purification Kit (Cell Guidance Systems, Cambridge, UK) according to the manufacturer’s instructions and subsequently purified by SEC (size-exclusion chromatography). Purified EVs were characterized by nanoparticle tracking analysis (NTA; NanoSight NS300, Malvern Panalytical, Malvern, UK; five video acquisitions; camera level 12–14; detection threshold 9–14) to determine size distribution and particle concentration, and by Western blotting to confirm the presence of the canonical exosomal marker CD9 (Bio-Rad, Hercules, CA, USA; MCA469). Calnexin (Santa Cruz Biotechnology, Dallas, TX, USA; sc-11397) was used as a negative control.

For Western blot validation, due to the low amount of protein obtained from vesicles, samples from cells cultured under normoxic or hypoxic conditions were pooled before extraction. Briefly, samples obtained using the Exo-spin™ Kit were lysed with 5× RIPA buffer (250 mM Tris-HCl, pH 7.4; 750 mM NaCl; 5% NP-40; 0.5% SDS; 2.5% sodium deoxycholate; 2 mM EGTA; 20 mM sodium fluoride; protease inhibitor cocktail P8849, Sigma-Aldrich, St. Louis, MO, USA) and run on a 13% polyacrylamide gel under non-reducing conditions. Proteins were then transferred to PVDF membranes and blocked for 2 h at room temperature (RT) with 10% bovine serum albumin diluted in TBS-Tween. Membranes were incubated overnight at 4 °C with primary anti-CD9 (1:2000) and anti-calnexin (1:500) antibodies. The membranes were then washed six times for 5 min each in TBS–Tween and incubated for 1 h at RT with HRP-conjugated secondary antibodies (1:15,000). The membranes were again washed six times in TBS–Tween and incubated for 5 min at RT with Clarity Western ECL Substrate (Bio-Rad Laboratories, Hercules, CA, USA). Proteins were visualized by briefly exposing the membranes to autoradiographic CL-XPosure Film (Thermo Fisher Scientific, Waltham, MA, USA). Densitometric analysis of Western blots was performed using the Analyze → Gels function of ImageJ version 1.54s.

### 2.4. miRNA Extraction and Quantification

Total miRNAs were extracted from purified EV preparations using the Maxwell^®^ RSC miRNA Plasma and Serum Kit (Promega, Cat. No. AS1680, Madison, WI, USA) according to the manufacturer’s instructions, employing the automated Maxwell^®^ RSC instrument (Promega, Madison, WI, USA) to ensure consistent yield and minimize manual handling. MiRNA concentration was assessed by fluorometric quantification using the Qubit™ microRNA Assay Kit (Thermo Fisher Scientific, Waltham, MA, USA). Purified miRNAs were immediately aliquoted and stored at −80 °C.

### 2.5. miRNA Library Preparation and Sequencing

The QIAseq miRNA Library Kit (QIAGEN N.V., Hilden, Germany) was used for library preparation following the manufacturer’s instructions. Final libraries were verified using both the Qubit 2.0 Fluorometer (Invitrogen, Carlsbad, CA, USA) and the Agilent Bioanalyzer DNA assay or Caliper (PerkinElmer, Waltham, MA, USA). After preparation, libraries were sequenced on the NovaSeq 6000 platform (Illumina, San Diego, CA, USA). Raw reads were first evaluated with FastQC [19], and reports were aggregated using MultiQC [20]. Adapters were removed using Cutadapt [21]. Following trimming, reads outside the 15–35 nucleotide range were excluded to enrich for mature miRNAs. A quality threshold of Phred score ≥ 20 was applied to minimize sequencing errors. Trimmed reads were aligned to the canine reference genome (CanFam3) using STAR [22], with parameters optimized for short RNA fragments. To ensure accurate quantification, alignments were restricted to annotated microRNA loci provided by miRBase [23]. Read counts per miRNA were obtained with featureCounts [24]. Multi-mapping reads were proportionally assigned to candidate loci, reflecting the repetitive nature of many miRNA genes.

### 2.6. Filtering, Normalization, and Differential Expression Analysis

Downstream analyses were performed in R v4.3.3 (R Core Team, Vienna, Austria, 2023) using edgeR [25] and limma [26]. To reduce noise from lowly expressed features, only miRNAs with counts per million (CPM) greater than 1 were retained. Library sizes were normalized using the trimmed mean of M values (TMM) method. Expression values were transformed to logCPM using the voom algorithm, which models mean–variance relationships.

### 2.7. Selection of Condition-Associated microRNAs

Condition-specific miRNAs were selected based on presence–absence patterns. Reads consistently detected in one condition (normoxia or hypoxia) but absent in the other were retained as biologically relevant. Venn diagram analyses were applied to highlight unique and overlapping miRNAs across conditions. Furthermore, curated lists of starvation- or hypoxia-associated miRNAs were retrieved from the literature to ensure that known regulators were incorporated into the candidate panel. This integrative approach allowed the identification of both computationally significant and biologically meaningful miRNAs.

### 2.8. Hierarchical Clustering and Heatmap Visualization

Normalized CPM values were filtered to retain miRNAs with total counts greater than 10,000 across all samples. Hierarchical clustering was then performed separately on samples and on miRNAs using complete linkage and Euclidean distance (factoextra/cluster package, R v4.3.3). Dendrograms were generated to assess clustering structure, and samples were annotated according to biological condition (normoxia vs. hypoxia). The top 20 most variable samples were selected for in vitro validation.

### 2.9. Real Time PCR Quantification

Forty-three selected miRNAs differentially expressed based on RNA-seq data (Table 1) were validated by qPCR using the miRCURY LNA miRNA PCR assay (Qiagen, Hilden, Germany). Two internal controls (spike-ins) were provided by the kit, while three housekeeping miRNAs (Cfa-miR-30e, Cfa-miR-27a, and Cfa-miR-301a) were used to normalize qPCR data. These were selected from RNA-seq data by filtering for miRNAs with unchanged logCPM across both cell lines and experimental conditions. The experimental design and gene expression analysis were performed using GeneGlobe system analysis provided by Qiagen (https://geneglobe.qiagen.com/us/analyze, accessed on 12 April 2026). miRNA fold regulation (hypoxia vs. normoxia) was calculated as 2^−ΔΔCq^, and *p*-values were calculated based on a Student’s *t*-test.

### 2.10. Pathway Enrichment Analysis

Predicted target genes for each validated miRNA were identified using miRDB, a machine learning algorithm trained on high-confidence experimental data [27]. For each miRNA, the top-ranked predicted targets with a target score ≥ 80 were extracted from miRDB. The compiled list of target genes was then uploaded to DAVID bioinformatic software (DAVID Knowledgebase v2025_1) an interactive gene-set enrichment analysis tool, to identify enriched Gene Ontology (GO) terms. Enrichment results were corrected for multiple testing, and only pathways with FDR < 0.05 were considered.

## 3. Results

### 3.1. Extracellular Vesicle Characterization

**Figure 1:**
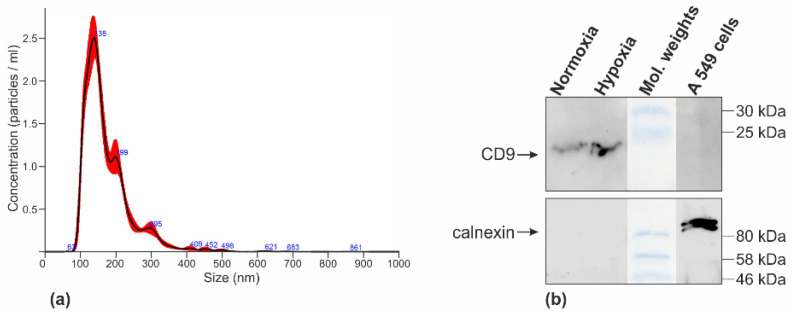
NTA revealed EVs with mean diameters of 155–174 nm and concentrations ranging from 10^9^ to 10^10^ particles/mL. EVs obtained from Wall cell line in hypoxic condition (**a**). Western blot analysis of EV lysates confirmed the presence of canonical exosomal marker (CD9) (top image) and the absence of calnexin (bottom image). A549 cellular lysate was used as positive control for calnexin (**b**).

Nanoparticle tracking analysis confirmed that most of the particle population was within the expected size range for small extracellular vesicles. Across all samples and conditions, particle sizes ranged predominantly between ~75 and 300 nm, with mean diameters between 172.1 ± 2.4 nm and 194.7 ± 1.4 nm, mode diameters between 112.8 ± 3.0 nm and 146.4 ± 11.2 nm, and concentrations between 10^9^ and 10^10^ particles/mL, consistent with the expected size and abundance of small extracellular vesicles. Western blot analysis of EV lysates confirmed the presence of the canonical exosomal marker CD9, while the endoplasmic reticulum marker calnexin was absent, supporting the absence of cellular contamination. Representative EVs obtained from pooled Wall, D22, Penny, and D17 cell lines under normoxic and hypoxic conditions were confirmed by Western blot for the exosomal marker CD9. No significant differences in EV size distribution or yield were observed between cell lines or between hypoxic and normoxic conditions. Figure 1 shows a representative NTA analysis of the Wall cell line under hypoxic conditions and a Western blot demonstrating EV isolation.

### 3.2. miRNome Profiling by RNA-Seq Analysis

**Figure 2:**
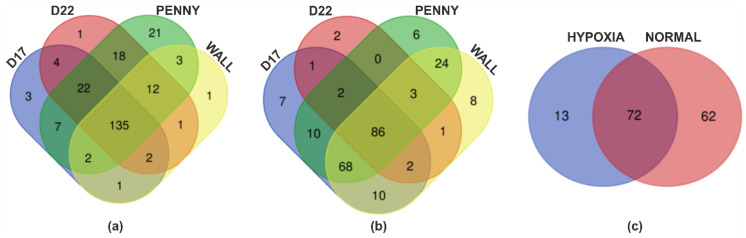
VENN analysis comparing all miRNA expressed in normal condition showed that 135 miRNA were present in all analyzed cell lines (**a**). VENN analysis comparing all miRNA expressed in hypoxia condition showed that 86 miRNA were present in all analyzed cell lines (**b**). VENN analysis showing all miRNA exclusively expressed in hypoxia (*n* = 13) and in normoxia (*n* = 62) in all cell lines (**c**).

**Figure 3:**
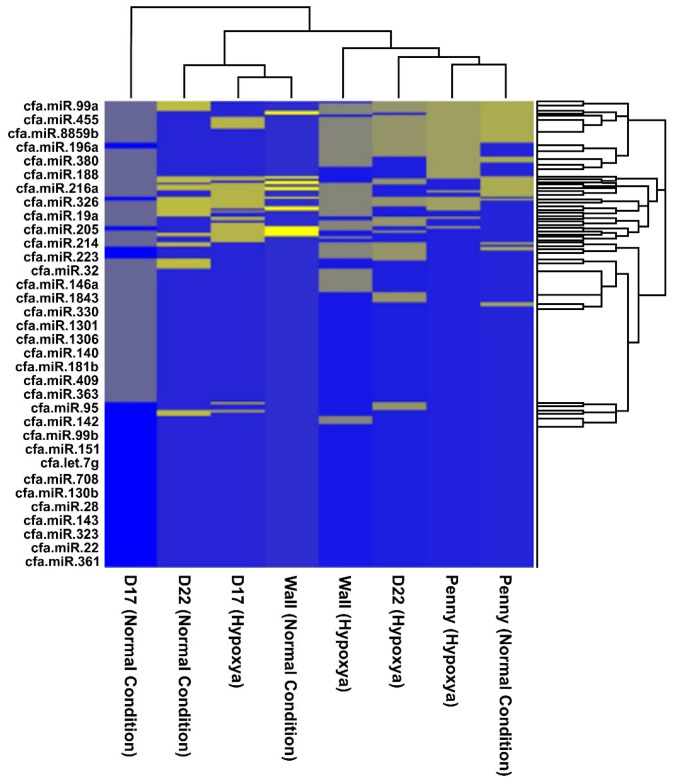
Hierarchical Cluster depicting the different levels of miRNA expression under nomoxic and hypoxic conditions in D17, D22, Wall and Penny cell lines. Expression values, derived from normalized log-CPM data, are represented on a continuous color scale ranging from yellow (high expression) through grey (intermediate expression) to blue (low or absent expression).

miRNome profiling identified 233 miRNAs expressed in EVs from all analyzed cell lines (cut-off logCPM > 0, or equivalently CPM > 1). Venn diagram analysis revealed that 135 miRNAs were present under normoxic conditions, while 86 miRNAs were detected under hypoxic conditions. 13 miRNAs were uniquely expressed under hypoxia, while 62 were unique to normoxia (Figure 2a–c). Hierarchical clustering confirmed distinct grouping of normoxic and hypoxic samples, demonstrating a slight hypoxia-dependent modulation of EV-miRNA cargo, more evident in the D22 and Wall cell lines. Bioinformatic filtering and hierarchical clustering identified 20 miRNAs (Figure 3). Based on these results, the D22 and Wall cell lines were selected for miRNA validation by qPCR. A total of 43 miRNAs were validated, with 22 selected from RNA-seq analysis (*n* = 12 from Venn analysis and *n* = 10 from hierarchical clustering). An additional 21 miRNAs were included based on literature data regarding the biological effects of hypoxic stimuli in vitro (Table 1).

### 3.3. Validation of Selected miRNAs

qPCR analysis of Wall and D22 cell lines showed that, in the Wall cell line, miR-222-3p and miR-186-5p were significantly downregulated under hypoxic conditions (*p* ≤ 0.05). In contrast, no significant differences were observed in the D22 cell line (Table 2). No significant changes were detected for the other miRNAs analyzed.

### 3.4. Functional Assay and Pathway Analysis of miR-222-3p and miR-186-5p

miRDB analysis identified *n* = 171 predicted target genes for miR-222-3p (Supplementary File S1) and *n* = 547 predicted target genes for miR-186-5p (Supplementary File S2). DAVID pathway enrichment analysis (FDR < 0.05) did not reveal any significant pathways for target genes regulated by miR-222-3p (Supplementary File S3), whereas a significant enrichment in various cancer-related and signaling pathways was observed for miR-186-5p (Supplementary File S4). The top enriched pathways included protein serine/threonine kinase activity, intracellular signal transduction, and zinc ion binding, which are well-known mediators of tumor cell proliferation, differentiation, and survival (Table 3).

## 4. Discussion

The content of tumor-derived EVs represents a potential source of tumor biomarkers [28,29], which may be used as diagnostic or prognostic markers, as well as therapeutic targets [30,31]. In OSA, increasing evidence indicates that EV-associated miRNAs reflect tumor aggressiveness and metastatic potential, supporting their relevance as minimally invasive biomarkers. Recent studies suggest that tumor-derived EVs may modulate tumor growth and/or metastasis either positively or negatively [32,33], highlighting their active role in shaping the tumor microenvironment rather than acting as passive by-products of cancer cells.

Hypoxia is present in approximately 90% of solid tumors and is considered a hallmark of cancer. Through the activation of hypoxia-inducible factors, hypoxia promotes angiogenesis, metabolic reprogramming, invasion, and resistance to therapy [34]. We previously demonstrated that microenvironmental hypoxia is involved in canine osteosarcoma and that HIF-1α expression is significantly associated with worse prognosis [14]. Additionally, we demonstrated that OSA cell lines under CoCl_2_ treatment accumulate HIF-1α in the nucleus, inducing the transcription of specific hypoxia-related genes, suggesting effective biological effects of HIF-1α on miRNA regulation [17]. Based on these premises, we focused on the capability of canine osteosarcoma cell lines to release miRNA-containing EVs under chemically mediated hypoxic stimulus. This approach allowed us to investigate whether chemically induced hypoxia not only affects intracellular miRNA expression but also influences EV-associated miRNA cargo.

Here, we showed that canine OSA cell lines can release miRNA-containing EVs under normal conditions and that the miRNA signature was slightly associated with each OSA cell line analyzed and with experimental conditions. This observation is consistent with the known biological heterogeneity of osteosarcoma and suggests cell line–specific EV-miRNA profiles [35]. Bioinformatic analyses revealed that canine OSA cell lines released 233 EV-associated miRNAs common to all conditions examined. In addition, hypoxic stimulation reshaped the EV-miRNA landscape, with 86 miRNAs uniquely associated with hypoxic conditions. These findings suggest that hypoxia selectively modulates miRNA loading into EVs rather than eliciting a uniform transcriptional response.

Based on bioinformatic analysis, we focused on the validation of forty-three EV-associated miRNAs in the D22 and Wall cell lines. Among these, miR-222-3p and miR-186-5p were significantly downregulated in the Wall cell line, as confirmed by qPCR, while no significant downregulation was observed in the D22 cell line. The downregulation of miR-186-5p observed in hypoxic EVs is consistent with findings in human oncology, where low miR-186-5p levels enhance tumor angiogenesis via activation of the HIF-1α/PRKCA/p-ERK signaling pathway and regulate the expression of HIF-1α [36,37]. This supports the hypothesis that miR-186-5p may function as a conserved hypoxia-responsive miRNA, whose reduced EV-associated levels contribute to the establishment of a pro-angiogenic tumor microenvironment. Such a mechanism may favor tumor adaptation to oxygen deprivation and promote disease progression. These findings are also supported by pathway enrichment analysis of putative target genes of miR-186-5p involved in different cellular pathways, including protein interactions and molecular functions, while its role in human osteosarcoma remains unknown.

Similarly, we observed reduced expression of miR-222-3p in the Wall cell line. In canine cancer, miR-222-3p has been recently identified in plasma-derived EVs from dogs with lymphoma [12], while in human osteosarcoma, the exact role of miR-222-3p remains unclear. One study demonstrated that miR-222-3p promotes osteosarcoma cell proliferation and invasion by targeting TIMP3 mRNA [11], while another study reported that low levels of miR-222-3p in plasma are associated with poor prognosis, diagnosis, and chemosensitivity prediction [38].

Several studies have reported the pivotal role of miRNAs (including miR-1, miR-133b, miR-34, and miR-196) in canine OSA, underlining the importance of these molecules in the regulation of key oncogenic pathways [2,8,39]. In particular, the downregulation of circulating miR-214-3p is significantly associated with worse prognosis in canine OSA patients [40]. However, none of these miRNAs overlapped with the EV-associated miRNAs identified in the present study, suggesting that distinct regulatory mechanisms may govern circulating versus EV-encapsulated miRNAs under hypoxic conditions.

These findings suggest that EVs can be secreted under hypoxic conditions in vitro and, if also demonstrated in clinical samples, may act as dynamic mediators of tumor microenvironment adaptation, mirroring the oxygen-sensitive molecular reprogramming observed in human OSA. Given the strong biological and molecular parallels between canine and human diseases, the identification of hypoxia-responsive EV-associated miRNAs reinforces the translational value of canine OSA as a comparative oncology model. Moreover, the stability and detectability of EV-associated miRNAs in biological fluids support their potential use as minimally invasive biomarkers for monitoring tumor hypoxia, progression, and therapeutic response.

Although the present work provides proof of concept of chemically induced hypoxia-mediated modulation of EV-associated miRNAs in canine OSA, it remains limited to in vitro observations and to a single cell line. The use of chemically induced hypoxia and established cell lines may not fully recapitulate the complexity of the in vivo tumor microenvironment. Further studies using hypoxia chamber conditions and serum-derived EVs from affected dogs are required to validate these candidate miRNAs in vivo and to elucidate their mechanistic contribution to metastatic spread. Expanding the analysis to a broader panel of hypoxia-regulated miRNAs may also help identify species-specific regulatory networks and refine biomarker selection for translational applications.

## 5. Conclusions

Canine osteosarcoma cells under chemically induced hypoxic conditions release different EV-associated miRNA profiles compared to normoxic conditions. miR-186-5p and miR-222-3p were significantly downregulated in the Wall cell line, suggesting a role for EVs in tumor adaptation under hypoxic conditions. These preliminary findings provide a basis for further investigation into the effects of hypoxia in canine osteosarcoma and for evaluating EV-associated miRNAs as minimally invasive biomarkers.

## Figures and Tables

**Table 1:** List of miRNAs filtered by Venn Diagrams, Hierarchical Analysis, Literature Data and validated by miRCURY LNA miRNA PCR assay.

Venn *n* = 12	Hierarchical *n*= 10	Literature *n* = 21
Cfa-miR-150	Cfa-miR-330	Cfa-miR-125a
Cfa-miR-486	Cfa-miR-455	Cfa-miR-31
Cfa-miR-138a	Cfa-miR-223	Cfa-miR-148a
Cfa-miR-95	Cfa-miR-8859b	Cfa-miR-210
Cfa-miR-145	Cfa-miR-326	Cfa-miR-8859a
Cfa-miR-137	Cfa-miR-146a	Cfa-miR-8858
Cfa-miR-127	Cfa-miR-205	Cfa-miR-17
Cfa-miR-432	Cfa-miR-214	Cfa-miR-149
Cfa-miR-338	Cfa-miR-380	Cfa-miR-1306
Cfa-miR-142	Cfa-miR-223	Cfa-miR-196a
Cfa-miR-454		Cfa-miR-143
Cfa-miR-451		Cfa-miR-497
		Cfa-miR-590
		Cfa-miR-186
		Cfa-miR-493
		Cfa-miR-193a
		Cfa-miR-222
		Cfa-miR-362
		Cfa-miR-505
		Cfa-miR-34a
		Cfa-miR-196b

**Table 2:** Down-regulation of EV-associated miRNAs in Wall cell line under hypoxic conditions. Data was normalized against three housekeeping miRNAs (*p* ≤ 0.05).

miRNA ID	Fold Regulation (Hypoxia vs. Normoxia)	*p*-Value
Cfa-miR-222-3p	−2.29	0.0289
Cfa-miR-186-5p	−1.93	0.0411

**Table 3:** Functional Assay and Pathway analysis of Putative Target genes of miR-186-5p.

Category	Term	Count	%	*p*-Value	FDR
GOTERM_MF_DIRECT	protein serine/threonine kinase activity	28	5.19	0.00000359	0.00231
GOTERM_BP_DIRECT	intracellular signal transduction	27	5.01	0.00000972	0.01
GOTERM_BP_DIRECT	protein phosphorylation	28	5.19	0.0000189	0.013
GOTERM_MF_DIRECT	zinc ion binding	82	15.21	0.0000519	0.0167
SMART	S_TKc	28	5.19	0.0000649	0.0163
INTERPRO	Kinase-like_dom_sf	33	6.12	0.0000121	0.0105
INTERPRO	Ser/Thr_kinase_AS	22	4.08	0.0000707	0.0397
INTERPRO	Prot_kinase_dom	29	5.38	0.0000915	0.0397
INTERPRO	Znf_RING/FYVE/PHD	30	5.57	0.00000407	0.00706
UP_KW_MOLECULAR_FUNCTION	Serine/threonine-protein kinase	24	4.45	0.000089	0.00289
UP_KW_MOLECULAR_FUNCTION	Kinase	38	7.05	0.000149	0.00324
UP_SEQ_FEATURE	DOMAIN:Protein kinase	29	5.38	0.000196	0.018
UP_KW_DOMAIN	Zinc-finger	63	11.69	0.0000124	0.000285

## Data Availability

The original contributions presented in this study are included in the article/Supplementary Materials. Further inquiries can be directed to the corresponding authors.

## Supplementary Materials

The following supporting information can be downloaded at: https://www.mdpi.com/article/10.3390/ani16081265/s1. File S1: predicted target genes list for miR-222-3p. File S2: predicted target genes for miR-186-5p. File S3: pathway enrichment analysis for target genes regulated by miR-222-3p. File S4: pathway enrichment analysis for target genes regulated by miR-186-5p.
